# Successful treatment of multisite hemorrhage by several methods in brain metastasis of choriocarcinoma: A case report

**DOI:** 10.1097/MD.0000000000010794

**Published:** 2018-05-25

**Authors:** Zhen Ren, Li Yu, Mengnv Xie, Yiyi Liang, Fang Zhu, Rui Huang, Zhibang Zhang, Chun Fu

**Affiliations:** a Department of Obstetrics and Gynecology, the Second Xiangya Hospital; b Department of Anesthesia, Xiangya School of Medicine, Central South University, Changsha, Hunan, China.

**Keywords:** brain metastasis, choriocarcinoma, pulmonary hemorrhage, uterine artery embolization

## Abstract

**Rationale:**

Brain metastasis of choriocarcinoma is a highly malignant gestational trophoblastic neoplasia (GTN) and has a notoriously poor prognosis. Hemorrhagic choriocarcinoma lesions may lead to life-threatening conditions also. Treatment of brain metastases of choriocarcinoma with hemorrhage in multiple sites is very difficult in clinical practices. A patient has been successfully treated in our hospital, which provides as clinical references for this difficulty in treating brain metastases of choriocarcinoma with hemorrhage in multiple sites.

**Patient concerns:**

A 28-year-old patient with gravida 2, para 0 was admitted in our hospital for amenorrhea, vaginal bleeding, and lower abdominal pain.

**Diagnoses:**

The patient was diagnosed as choriocarcinoma FIGO stage IV and the score of the Prognostic Scoring Index modified by the WHO was 15.

**Interventions:**

The patient received multiagent chemotherapy (EMACO regimen) soon after the diagnosis of choriocarcinoma with brain metastasis. During the therapy, the patient was given 3 different methods of treatment for metastatic site hemorrhage. An emergency surgery was performed to control massive bleeding from the metastatic lesions of broad ligament. Blood transfusions were given to treat acute left pulmonary hemorrhage. Uterine artery embolization (UAE) was performed to treat increased uterine bleeding.

**Outcomes:**

The patient achieved remission after 9 cycles of chemotherapy. She has been followed up for 14 months with no signs of tumor recurrence.

**Lessons:**

The diagnosis of choriocarcinoma may be difficult, especially in the setting with the limit of medical resources. The application of various diagnostic techniques such as x-ray, computed tomography, and magnetic resonance imaging is helpful for evaluating the patient's condition.

## 1 Introduction

Choriocarcinoma is a highly invasive tumor derived from trophoblast cells, which is easy to metastasize via the hematogenous pathway to multiple organs.^[1]^ The choriocarcinoma lesion has a high chance of bleeding due to high vascularity and affinity of trophoblast for blood vessels.^[2]^ These hemorrhage sites can be seen in many regions of the body, such as uterus, vagina, lung, kidney, brain, liver, intestines, and spleen.^[3–8]^

Cure rate of choriocarcinoma is up to 90% because of chemosensitivity, but choriocarcinoma lesion hemorrhage can be life-threatening. Selection of the treatment for hemorrhage sites is mainly based on the patient's general condition and the severity of the bleeding.^[9]^ Surgical way, arterial embolization, and radiotherapy with combination chemotherapy are proven as effective methods to stop bleeding.^[3–8]^ The treatment of choriocarcinoma hemorrhage has been improved, but multisite hemorrhage is still very difficult and may suggest poor prognosis. A particularly serious case is the choriocarcinoma syndrome, which consists of hemorrhagic manifestations of metastases in advanced germ cell cancer containing large elements of choriocarcinoma.^[10]^ Six such cases in 13 reported cases have a fatal outcome.^[10–12]^

About 10% to 28% of choriocarcinoma metastasize to the brain, and cerebral metastasis is a poor prognostic factor.^[2]^ Treatment of brain metastases of choriocarcinoma with multisite hemorrhage is very difficult in clinical practices. Accurate and timely treatments are very important for this critically ill patient, but there are very few reports of successful treatment. A patient has been successfully treated in our hospital, which may provide as clinical references for this difficulty in treating brain metastases of choriocarcinoma with multisite hemorrhage.

## 2 Case review

**Figure 1:**
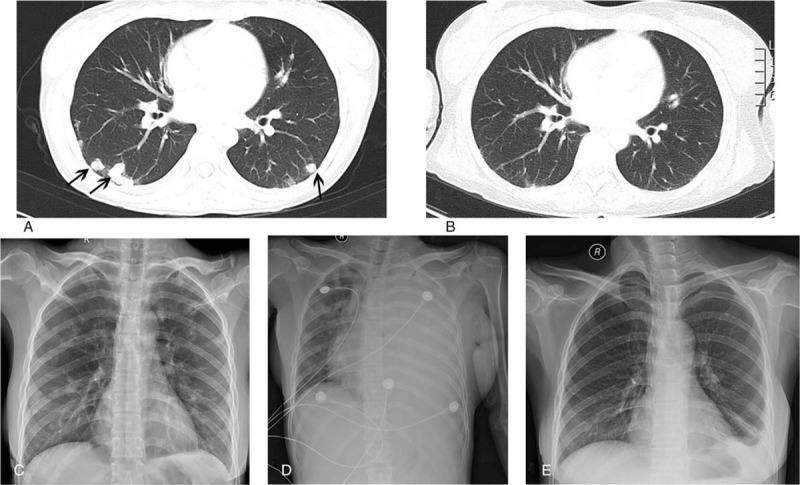
Lung computed tomography (CT) images and chest radiographs of the patient at different stages of treatment. (A) Multiple nodules of bilateral pulmonary were shown on CT image before chemotherapy (black arrows, metastatic nodules). (B) CT image revealed an almost normal lung field after treatment. (C) Multiple lung nodules were seen in bilateral lung field before treatment. (D) Large-scale pleural effusion in left lung field indicated massive hemorrhage of lung metastasis on the first day of the second chemotherapy. (E) Only little pleural effusion was in left lung field after treatment.

**Figure 2:**
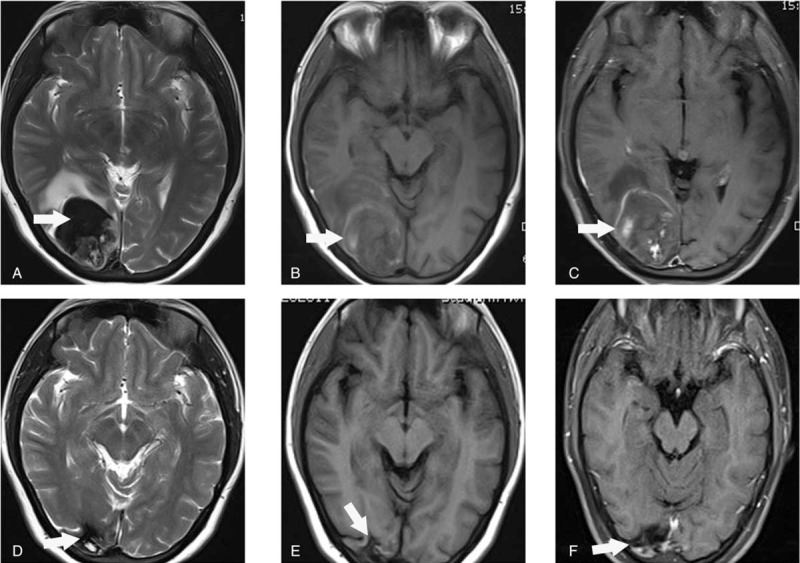
Brain magnetic resonance (MR) images of the patient before and after therapy. (A–C) before chemotherapy; (D, E) after 9 courses of EMA-CO. (A) A brain metastatic lesion was showed on the temporal, parietal, and occipital lobes of right hemispheres. Axial T2-weighted image (T2WI) of the lesion displayed heterogeneous low-signal intensity (white arrow). (B) The metastatic lesion (the same lesion in A) was slightly hyperintense on axial T1-weighted image (TIWI) (white arrow). (C) The signal of partial lesion showed enhancement on contrast-enhanced T1WI (the same lesion in A, white arrow). (D, E, and F) were T2WI, T1WI, and contrast-enhanced T1WI of the lesion after treatment (white arrows), respectively. The size of the lesion after treatment was significantly decreased compared with that before treatment.

**Figure 3:**
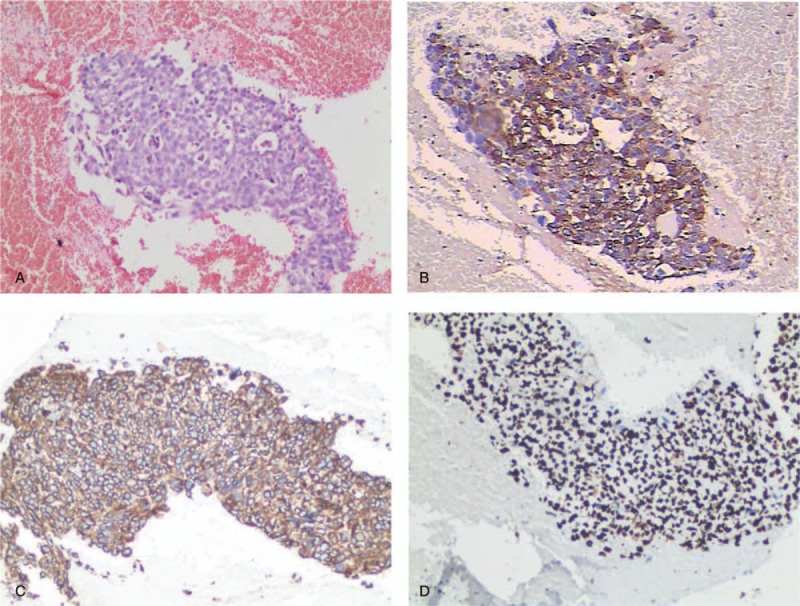
Pathological pictures of uterine cavity tissue in the patient. (A) Blood clot mixed with a small number of dysplasia trophoblastic cells and lack of villus structure were showed in tissue (hematoxylin and eosin staining, ×100). (B) The positive expressions of human chorionic gonadotropin (hCG) were found in dysplasia trophoblastic cells (immunohistochemical staining, ×100). A majority of trophoblastic cells showed positive expressions of cytokeratin (CK) and Ki-67 proteins, respectively, in C and D (immunohistochemical staining, ×100).

A 28-year-old woman (gravida 2, para 0) was admitted to our hospital with a complaint of amenorrhea with irregular vaginal bleeding for 3 month, and lower abdominal pain for 4 days. Her menstrual cycle was regular and the recent abortion was 1 year ago. Bimanual examination revealed the uterus was obviously larger than expected. Pelvic ultrasound showed a 12.6 × 11.5 × 9.1 cm size of uterine body and a complex echo mass with mixed cystic-solid appearance in the uterine cavity. The blood level of β-human chorionic gonadotropin (β-HCG) was 69,695 miu/mL (normal value, <3.0 miu/mL). The patient's primary diagnosis was hydatidiform mole and she received an emergency uterine curettage for significant vaginal bleeding. However, intrauterine content showed no hydatidiform mole tissue after suction curettage. The patient soon happened uncontrolled massive vaginal hemorrhage. The operation was immediately stopped and a Foley balloon catheter (injected 60 mL saline) was placed in the uterine to control bleeding effectively. Further detail examinations were performed soon after uterine curettage. Chest computed tomography (CT) scans and X-ray examination both identified lung metastatic foci (Fig. 1A, C). Magnetic resonance imaging (MRI) revealed brain metastases (Fig. 2A–C). Pathologic results were a small amount of trophoblastic cells and no villus in blood clot (Fig. 3A). The dysplasia trophoblast cells showed positive expressions of HCG by immunohistochemical methods (Fig. 3B). A majority of these trophoblast cells also have positive expressions of cytokeratin (CK) and nuclear-associated antigen Ki67 (Ki67) (Fig. 3C, D). On the basis of clinical and laboratory investigations, the patients were diagnosed as choriocarcinoma FIGO stage IV and the score of the Prognostic Scoring Index modified by the WHO was 15.

The patient received EMACO (etoposide, methotrexate, dactinomycin, vincristine, and cyclophosphamide) chemotherapy regimen the second day after curettage. The regimen was as follows: methotrexate 100 mg/m^2^, followed by methotrexate 200 mg/m^2^ over 12 hours day 1, etoposide 100 mg/m^2^ and dactinomycin 0.5 mg days 1 and 2, calcium folate 15 mg intramuscular injection every 12 hours days 2 and 3, alternating with cyclophosphamide 600 mg/m^2^ and vincristine 2 mg day 8, every 14 days. The patient received nine courses of EMACO chemotherapy. On the fifth day of the first course, the patient suddenly felt sharp and persistence abdominal pain, her face looked pale, and soon shocked. An emergency surgery was performed considering intraperitoneal hemorrhage. Three liters of blood and a lot of blood clots were accumulated in pelvic and abdominal cavity. The size of the enlarged uterus was the size of more than 4 months pregnant and multiple purple blue nodules appeared on the surface of uterine. The ruptured nodules were found in the left broad ligament and had been repaired quickly. So, the patient's vital signs were gradually recovered by active treatment. However, the patient experienced nausea and vomiting 5 days after operation. The symptoms disappeared by intravenous drip of Mannitol to reduce intracranial pressure. The subsequent neurological examinations showed she had no cerebral hernia.

On the first day of the second EMACO chemotherapy, the patient emerged with left-sided anterior chest pain accompanied by sweating and pale. The symptoms were slightly relieved without intervention after 30 to 40 seconds. But the patient still felt nausea and shortness of breath. Physical examination revealed no obvious breath sounds on auscultation of the left lung field. Chest X-ray indicated lung hemorrhage (Fig. 1D). The patient received timely blood transfusions and continued chemotherapy to reduce tumor load. The symptoms were gradually relieved after the third chemotherapy course. Intrathecal injections of methotrexate (15 mg every time, 2-week interval) were given at chemotherapy interval. The patient received 4 times of intrathecal methotrexate.

**Figure 4:**
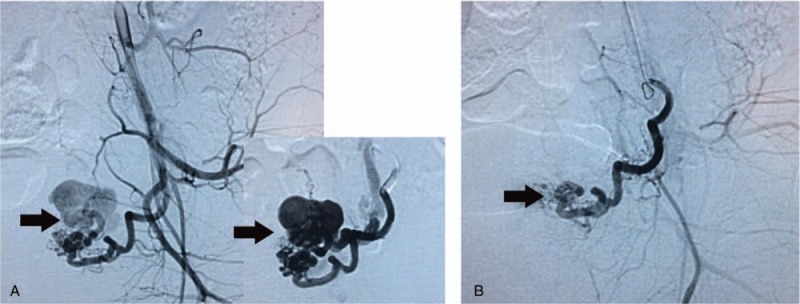
The pictures of uterine artery angiograms (UAE) for the patient. (A) Selective UAE proved active bleeding of the branches of uterine arteries before operation (black arrows). (B) Hemorrhage of blood vessels was ceased immediately after selective embolization (black arrow).

Serum β-HCG was decreased to normal after the 6th chemotherapy. It took 4 months for β-HCG to become normal. Changes in laboratory tests and imaging data are presented in Table 1. The patient then completed 3 consolidation courses of chemotherapy. The results of lung CT, brain MRI, and chest radiograph after treatments all showed favorable therapeutic response (Figs. 1B, E and 2D–F). Contrary to therapeutic effect, the amount and times of uterine bleeding increased gradually. In order to preserve fertility, uterine artery embolization (UAE) was performed to selectively embolize the bleeding vessel and successfully control uterine hemorrhage (Fig. 4). The patient has been followed up for 14 months at the outpatient clinic. She complained of no self-conscious discomfort and her menstruation returned to normal. The β-HCG value has been maintained in the normal range.

**Table 1:**
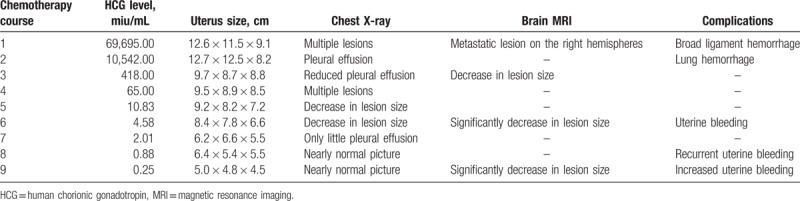
Laboratory tests and imaging data of the patient during the treatment.

The study was approved by the Second Xiangya Hospital of Central South University. Informed consent form was obtained from the patient.

## 3 Discussion

Choriocarcinoma is a highly malignant gestational trophoblastic neoplasia (GTN). Diagnosis of choriocarcinoma may be difficult in the setting with the limit of medical resources, where doctors made diagnosis on the basis of the clinical symptoms with an abnormal high level of serum β-HCG.^[1]^ It is very important to assess the patient's condition by a variety of diagnostic techniques such as X-ray, CT, and MRI. Choriocarcinoma patients need to receive chemotherapy as soon as the diagnosis is made. The choice of treatment regimen depends on the stage of the tumor and prognostic score. Our patient had been treated with EMACO regimen, which is the first-line chemotherapy regimen in GTN patients with a high risk.^[11]^ Our successful treatment also confirmed the effectiveness of the proposed scheme in patients with brain metastasis of choriocarcinoma.

Due to the characteristics of trophoblast cell growth, hemorrhage of choriocarcinoma lesions is easily seen during the whole therapy, even at the end of treatment.^[2]^ This case reflects the distinguishing feature of hemorrhage in multiple sites such as uterus, abdominal cavity, and pulmonary. The characteristic signs and symptoms of choriocarcinoma patients with different hemorrhagic sites have been described in previous literature. Patients with cerebral hemorrhage can present with headache, vomiting, dysarthria, and hemiparesis.^[5]^ Patients with pulmonary hemorrhage can suffer from hemoptysis and chest pain.^[10]^ Patients with vaginal bleeding can show massive vaginal hemorrhage, suprapubic pain, and even urinary retention.^[13]^

Conservative treatment for choriocarcinoma hemorrhagic lesions is first considered due to chemosensitivity. A case of metastatic testicular choriocarcinoma complicated by pulmonary hemorrhage was gradually relieved by chemotherapy combined with intensive supportive care.^[11]^ Arterial embolization has been proved to be an effective nonsurgical way of ceasing the bleeding. Transcatheter angiographic embolization is an accepted treatment modality to control recurrent massive genital bleeding and may be alternative to hysterectomy.^[14]^ Two cases of post-term choriocarcinoma with liver metastases complicated by profuse life-threatening hemorrhage were successfully treated by transcatheter angiographic embolization of the hepatic artery.^[6]^ In our case, brain metastasis of choriocarcinoma associated with 3 other parts of bleeding increased the difficulty of treatment. The patient was treated with EMACO chemotherapy and symptomatic treatment. The efficiency of conservative treatment was proved in controlling the pulmonary hemorrhage. We also succeeded in the treatment of uterine bleeding with conservative UAE.

Chemotherapy is the most fundamental and effective method for choriocarcinoma treatment and conservative treatment is first considered in complication therapy, but patients with a large amount of bleeding or uncontrolled bleeding may still require emergency surgery. Hysterectomy can be offered to patients with uncontrolled uterus bleeding.^[3]^ Decompression craniectomy and lesion resection of tumor were performed to treat choriocarcinoma with intracerebral hemorrhage.^[5]^ Exploratory laparotomy was applied in shock case induced by intraperitoneal hemorrhage.^[15]^

In conclusion, we present a case of brain metastasis of choriocarcinoma with multisites hemorrhage. During the therapy, the patient was given 3 different methods of treatment for metastatic site hemorrhage. These methods can improve the survival of patients.

## Author contributions

**Data curation:** Mengnv Xie, Yiyi Liang, Rui Huang, Chun Fu.

**Resources:** Li Yu, Chun Fu.

**Software:** Zhen Ren, Fang Zhu, Zhibang Zhang.

**Writing – original draft:** Zhen Ren, Chun Fu.

**Writing – review & editing:** Zhen Ren, Chun Fu.
